# The Constitution of the B.M.A.

**Published:** 1913-09-13

**Authors:** Chas. Buttar


### The Constitution of the B.M.A.

*To the Editor of* The Hospital.

Sir,—Though I cannot lay claim to the able replies to Dr. Fothergill's letters which have appeared in your Journal, I may be allowed, perhaps, now that Dr. Fothergill has ceased to ask questions, to give my reasons for the faith that is in me. I have been mixed up a good deal in the Insurance Act controversy; and, as a member of the Council of the British Medical Association during the critical period last year, I became appalled at the hopelessness of expecting any business-like action from that body. I am convinced that the whole trouble lay in the impossible "constitution" which has been developed in the course of years; and I have convinced myself, at all events, that no such reform in that constitution can be produced within any reasonable space of time as will allow of the adoption of business-like methods. I have watched constitution-tinkering going on year after year, but to me at least the actual results in benefits to the profession have not seemed commensurate with the time, money, and labour expended. And then came the great collapse, from which the profession and the Association have not yet recovered; so that we see now all sorts of local units up and down the country scrambling to make whatever terms they can for themselves. Take, for instance, the question of those green tickets for migrants. In the face of this mighty body, "eighty years old, acknowledged by the Government, etc., etc., with a membership of 20,000"—I thought it was nearer 25,000 last year—in face of this Association the authorities are at their old game, and the medical men of health resorts are being intimidated by threats that their territories will be invaded by Government nominees if they do not accept those green vouchers.

As a result of my experience I am compelled to the view that salvation lies in one direction alone; and that is the formation of a new body working on different lines, but with the approval and assistance of the British Medical Association, and taking to itself all the powers available at the present time. In the National Medical Guild is to be found this new and active body, ready to take up any case of fresh insult to the medical profession, possessed of such powers, tried or untried, as are allowed to a trade union, and willing to work not as a rival but as the coadjutor of the British Medical Association.

To my mind this is the answer, and the complete answer, to all Dr. Fothergill's questions. It is a matter of regret to me that it is impossible for the Association ever to become such a body itself. The report of its own Council showed this only too plainly; and I fear that all Dr. Fothergill's beautiful perorations will not alter hard unchangeable facts.—I remain, Sir, yours faithfully,

Chas. Buttar.

